# Heparan sulfate as a central modulator of growth factor dynamics in tissue regeneration

**DOI:** 10.1063/5.0289409

**Published:** 2025-11-13

**Authors:** Keyu Tao, Ali Shokoohmand, Simon M. Cool, Raymond A. A. Smith

**Affiliations:** School of Chemical Engineering, The University of Queensland, Brisbane, QLD 4072, Australia

## Abstract

Growth factors (GFs) are potent regenerative proteins that modulate biological responses and stimulate tissue repair by activating cell signaling pathways to enhance proliferation, differentiation, and migration. However, the rapid degradation and diffusion characteristics of GFs necessitate the use of supraphysiological doses and/or frequent administrations to maintain therapeutic effects, which can result in reduced efficacy and an increased risk of adverse events. To address these challenges, novel GF delivery systems seek to preserve bioactivity and modulate GF release, ultimately promoting more effective tissue regeneration. Heparan sulfate (HS) is an essential regulator of GF activity, executing molecular recognition and information storage for controlling extracellular matrix remodeling and cellular behavior during tissue development. In this perspective, we summarize the diverse roles of HS in tissue repair, focusing on its interactions with GFs. These include protective functions, the tonic release of GFs, promoting the complexation of GFs with their cognate receptors, and enhancing the activation of intracellular signaling. Finally, we provide perspectives on using HS as a component of novel biomaterials and medical devices for improving tissue regeneration.

## I. BACKGROUND

Increasing life expectancy and rapidly ageing populations result in an increasing prevalence of chronic wounds, diseases, and non-fatal injuries. It is estimated that 1%–2% of the world's population will suffer chronic wounds during their lifetime,^1^ causing significant global disease and economic burden. The global wound care market is projected to grow from USD 21.5 × 10^9^ in 2023 to approximately USD 28.6 × 10^9^ in 2028.^2^ Despite the availability of new biopharmaceutical formulations, wound dressings, and other new clinical solutions in recent decades, satisfactory tissue regeneration remains elusive. In many cases, the injured site fails to achieve complete re-epithelialization, and tissue function is not fully restored, particularly in severe injuries that require surgical intervention. Thus, there is great interest in developing advanced regenerative therapies that fully restore tissue function.

Particular attention has been given to closely mimicking the extracellular microenvironment to regulate signal transduction pathways in a controlled manner, facilitating better tissue repair. Growth factors (GFs), key signaling molecules in the extracellular matrix (ECM) and pericellular environment, show promising regenerative effects in preclinical models by enhancing cell proliferation, migration, and differentiation. In rodent models, platelet-derived growth factor-BB (PDGF-BB) can trigger granulation tissue formation and stem cell proliferation, while vascular endothelial growth factor (VEGF) is vital for angiogenesis.^3,4^ Multiple GF-based medical products have been approved by the Food and Drug Administration (FDA) for topical administration and direct injections, such as Infuse^®^ Bone Graft [bone morphogenetic protein-2 (BMP-2) based], PELNAC^®^ [fibroblast growth factor-2 (FGF-2) based], and Citrix^®^ CRS [transforming growth factor beta 1 (TGF-β1) based], most of which are involved in angiogenesis and osteogenesis.^5^

Although GFs have shown significant therapeutic potential, the inherent properties of proteins in physiological conditions are obstacles to their successful translation into the clinic. Major limitations include thermal instability, short half-life, rapid diffusion from target sites, deactivation/hydrolysis by proteases, and cellular internalization, all of which lead to suboptimal efficacy.^6,7^ For instance, the half-life of BMP-2 *in vivo* is as short as 7 min.^8^ To achieve the therapeutic benefit, high or multiple doses of GFs are required, which frequently exceed physiological levels, leading to high costs and potential side effects, including tumorigenesis and ectopic tissue formation.^9^ Thus, there is a strong interest in developing methods for controlled, precise, sustained, and localized release of GFs to an injury site in a spatiotemporal manner with minimal side effects. Two common approaches are protein engineering and the development of novel GF delivery systems. In the first approach, GFs are engineered to improve stability, half-life, and binding ability for receptors/co-receptors at the cell surface. The second approach involves developing appropriate delivery methods that mimic the natural tissue microenvironment, inspired by the controlled release mechanisms found in the ECM.^10^ Heparan sulfate (HS), a subclass of glycosaminoglycan (GAG), serves as a co-receptor and GF sink, improving protein stabilization, maintaining sustained GF release, and enhancing receptor binding. This, in turn, extends the half-life and activity of GFs, orchestrating effective tissue repair and regeneration.

**FIG. 1.**
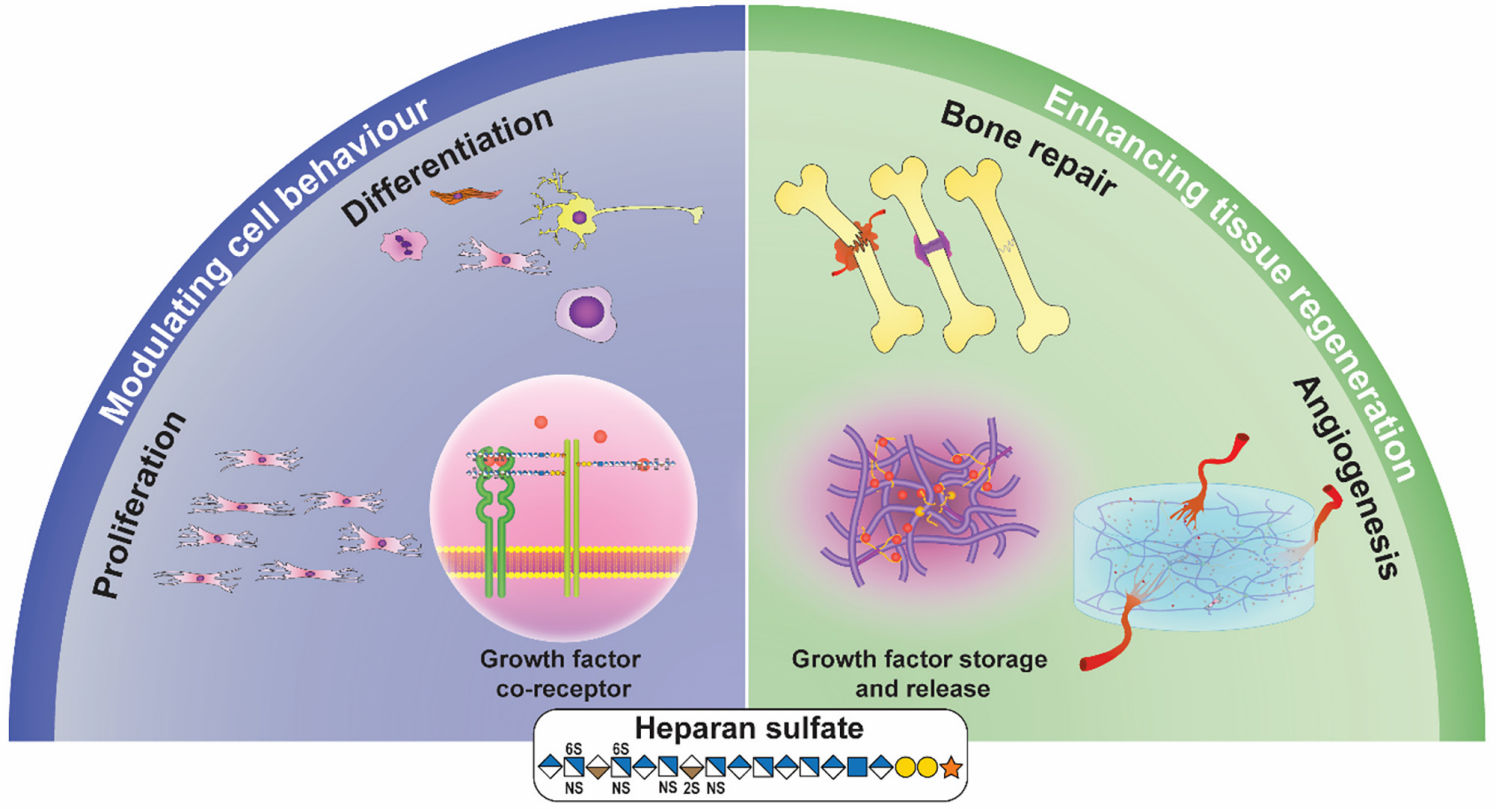
The roles of HS in modulating growth factors in tissue repair. HS influences cell behavior through its functions as a co-receptor, enhancing proliferation and differentiation. These events are critical in tissue repair processes, such as angiogenesis, or in healing large injuries, such as broken bones. The monosaccharides represented in this figure are derived from the symbol nomenclature for glycans (SNFG).^11^

In this perspective, we will discuss the essential roles HS plays in tissue regeneration through interactions with GFs, summarized in Fig. 1. First, we focus on the ability of HS to improve the stability of multiple GFs. The second section highlights the local and sustainable delivery of GFs to cells by HS. Third, we discuss how HS increases GF interactions and affinity to cognate receptors and enhances cellular signaling by acting as a co-receptor. Finally, we discuss how HS regulates multiple GF signaling pathways that control cellular proliferation, aging, and potency.

## II. HEPARAN SULFATE

### A. HS biosynthesis, structure, and function

**FIG. 2.**
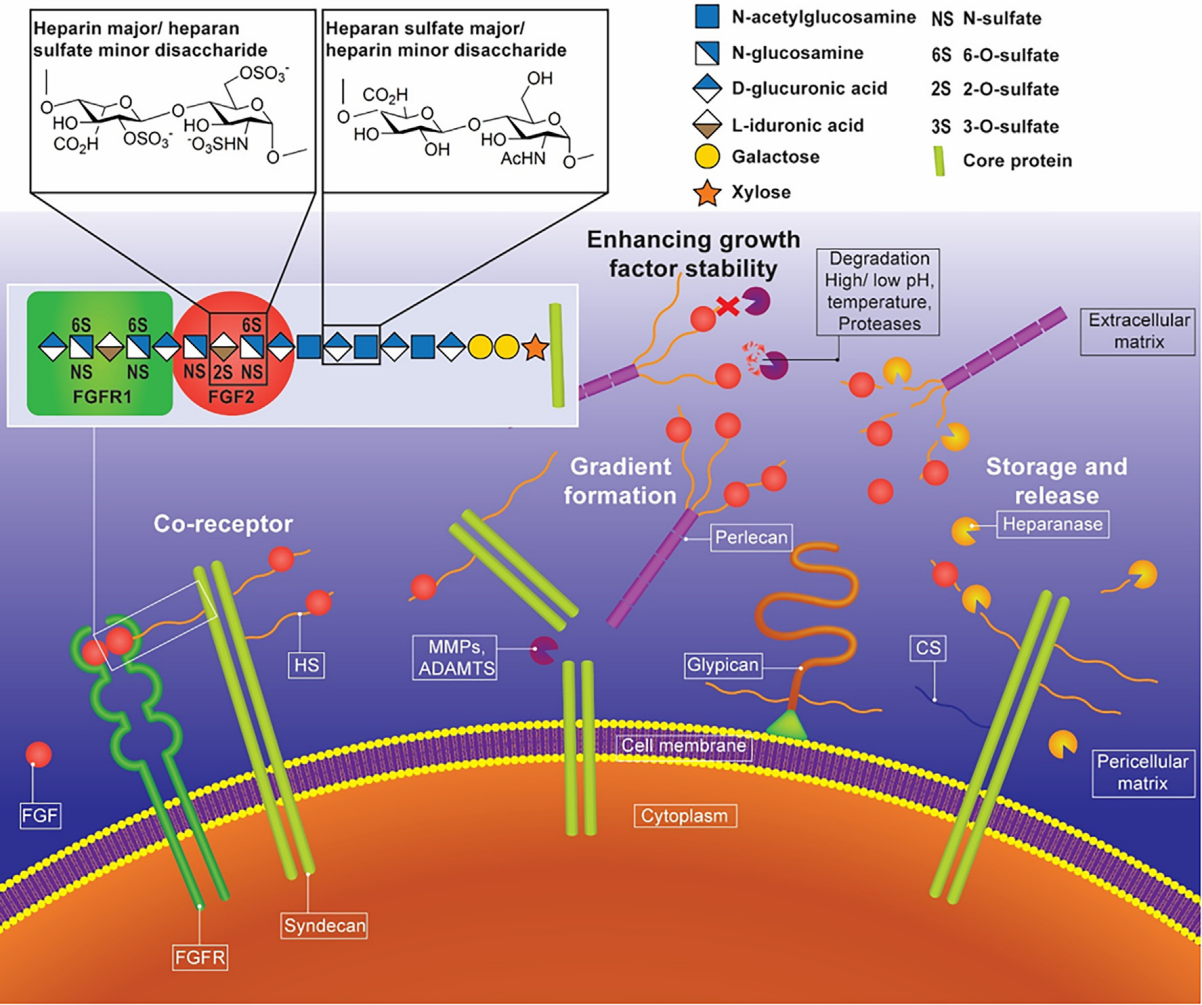
The functions of HS in biological systems. Interactions of HS and growth factors, preserving and stabilizing GFs in the ECM, regulating the availability of GFs to cells, and facilitating GF binding to its cognate cell surface receptor. The monosaccharides represented in this figure are derived from the symbol nomenclature for glycans (SNFG).^11^

HS is expressed ubiquitously in almost all mammalian cells and tissues, at the cell membrane and throughout the ECM, and exerts various biological functions in a tissue-specific manner. It is a linear polysaccharide composed of up to 100 repeating disaccharide units of α1,4-linked uronic acid and glucosamine residues, variably modified with sulfate groups. HS chains exist covalently bound to serine residues on a protein core, termed heparan sulfate proteoglycans (HSPGs, Fig. 2). HSPGs exist in several categories based on their location, including cell membrane HSPGs (e.g., syndecans and glypicans), secreted ECM HSPGs (e.g., perlecan), and secretory vesicle PGs (serglycin).^12^ The structure and abundance of HS are strictly regulated and dependent on the stage of development and injury.^13^ For example, in the developing lung, the mesenchyme at the sites of prospective budding expresses HS low in *O*-sulfate content, while the basement membrane of branching epithelial tubules contains highly sulfated HS chains.^14^ Injury can induce alterations in the sulfation and expression patterns of HSPGs.^15^ HS is essential throughout life, from guiding embryonic development to maintaining tissue homeostasis and influencing ageing.

The mechanism by which HS influences biological processes involves interactions with GFs. Negatively charged sulfate and carboxyl groups in HS chains enable interaction with proteins through electrostatic ion pairing with positively charged lysine, arginine, and histidine residues on protein surfaces. Occasionally, it may also be mediated by hydrogen bonding and van der Waals' bonds.^16^ Due to the high degree of structural heterogeneity in HS chains through variations in sulfation and acetylation patterns, chain length, and conformation, more than 400 HS-binding proteins have been identified.^17^ These include a range of GFs, neurotrophins, cytokines, chemokines, morphogens, ECM structural proteins, cell-adhesion molecules, proteases, and protease inhibitor proteins.^18^ Many GFs, which play essential roles in tissue repair, contain HS-binding domains, such as FGFs, PDGFs, epidermal growth factor (EGF), VEGF, and BMPs.^19–21^ The active HS–GF complex can be extracted upon proteolytic degradation of the ECM, further confirming HS–GF interactions *in situ*. The removal of cell surface HS by heparinase significantly perturbs the activation of these reparative factors, thereby delaying recovery.^22,23^

### B. HS-binding proteins

HS chains exhibit variable binding affinities for different GFs due to their vast structural diversity. We have previously observed considerable variability in the binding affinity of porcine mucosal HS to several GFs, including TGF-β1, BMP-2, FGF-2, PDGF-BB, and VEGF_165_, suggesting that some HS chains may be enriched in binding sites for one protein but lack binding sites for others.^24^ Even within one GF family, interactions between GFs and HS can vary significantly, as evidenced by using six FGFs from five subfamilies, suggesting FGF–HS interactions are molecule-specific.^25^ Structure–function relationships of HS are being uncovered to pinpoint the structural prerequisites for effective binding.^26–33^ Some of these findings are summarized in Table I.

**TABLE I.** Structural requirements for the binding of HS to several proteins essential in tissue regeneration.

Protein	Sulfation requirements (*N*-, 6-*O*, 3-*O*, 2-*O*)	Minimal/optimal oligosaccharide length (dp)	Source
FGF-1	*N*-, 6-*O*, 2-*O*	dp5–7 (minimal)	31, 34
FGF-2	*N*-, 2-*O*	dp5 (minimal), dp14 (optimal)	35–37
FGF-10	*N*-, 6-*O*	dp8	38
FGF-18	*N*-, 2-*O*	dp8	38
BMP-2	*N*-	dp4 (minimal), dp12 (optimal)	30
TGF-β1	*N*-, 6-*O*	dp4 (minimal), dp18 (optimal)	39
VEGF_165_	*N*-, 6-*O*	dp6–7 (minimal), dp14 (optimal)	32, 40
IL8	*N*-, 6-*O*, 2-*O*	dp6 (minimal), dp22–24 (optimal)	41
HGF	*N*-, 2-*O*	dp8	38

In this perspective, we will focus primarily on those proteins that have displayed therapeutic potential, specifically the FGFs, BMPs, and VEGF.

### C. Using heparin to inform HS: protein interactions

HS is an analog of heparin, a drug approved by the FDA and European Medicines Agency (EMA) for anticoagulation. Prior approval of a molecule of similar structure suggests that incorporating HS into materials designed for clinical use is highly feasible. Heparin and HS share the same biosynthetic pathway and comprise the same pool of disaccharide subunits. However, heparin and HS markedly differ in composition and function. Heparin is highly sulfated, with approximately 2.6 sulfates per disaccharide, while HS has approximately 0.6 sulfates per disaccharide.^42^ While markedly higher in sulfation, short heparin oligosaccharides are structurally analogous to the NS domains within HS chains, making these oligosaccharides a useful tool for studying GF interactions with direct relevance to HS. These oligosaccharides are easily prepared from unfractionated heparin through enzymatic depolymerization or acid hydrolysis and can be further desulfated to yield oligosaccharides of distinct sulfation patterns. Many of the structure:function studies listed in Table I utilize such oligosaccharides for elucidating the binding requirements of specific proteins to HS and heparin.

### D. The limitations of using heparin

Heparin is frequently used in tissue engineering for the sustained release of GFs from various materials due to the relative ease of procuring large quantities from multiple suppliers. Though widely studied, heparin is known to have numerous side effects due to high sulfation, leading to promiscuous binding to many proteins. Some well-known side effects include reduced bone density, osteoporosis, and thrombocytopenia.^28,43^ In addition, the potent anticoagulant activity of heparin is undesirable when the targeted reparative process involves clot formation through fibrin–thrombin interactions. Heparin is also known to produce variable effects on HS in certain circumstances, such as inhibiting GDF-5 and altering the molecular phenotype of human mesenchymal stromal cells (MSCs).^44,45^ Thus, HS, which is structurally more complex and lacks the side effects of heparin, offers a more promising candidate for delivering GFs for tissue regeneration. In recent years, HS has been applied in various tissue engineering applications to facilitate tissue development and repair.^46,47^

## III. HS AS A REGULATOR OF GF STABILITY, DIFFUSION, AND RELEASE

### A. HS and GF stability

The role of HS in stabilizing various GFs is well documented. Porcine mucosal-derived HS, affinity isolated against the FGF-2 heparin-binding domain, can protect FGF-2 from thermal denaturation, increasing its denaturation temperature by ∼6 °C.^48^ Short heparin oligosaccharides of dp10 and above have also been shown to protect FGF-1, -2, -7, -9, and -18 from thermal denaturation.^4,25^ In proteolytic wound environments, HS shields GFs from enzymatic degradation. Heparinase-treated HS leads to FGF-2 degradation, while exogenous HS and heparin inhibit MMP2 and protect FGF-2 from tryptic and chymotryptic digestion.^49–51^ Heparin has also been shown to preserve FGF-1 and FGF-2 activity after exposure to acidic stress,^52^ cryopreservation,^52^ and copper-catalyzed oxidation.^53^ Several studies have documented the effects of HS on the stability of BMP proteins. HS and heparin have both been shown to enhance the half-life of BMP-2 and protect it from thermal denaturation, preserving bioactivity.^54–56^ HS can protect VEGF_165_ from thermal degradation, plasmin digestion, and freeze–thaw cycles.^4^ Heparin-based star-PEG hydrogels have also been shown to protect IL-4 from thermal and proteolytic degradation.^4,57^

### B. Formation and regulation of GF gradients

**FIG. 3.**
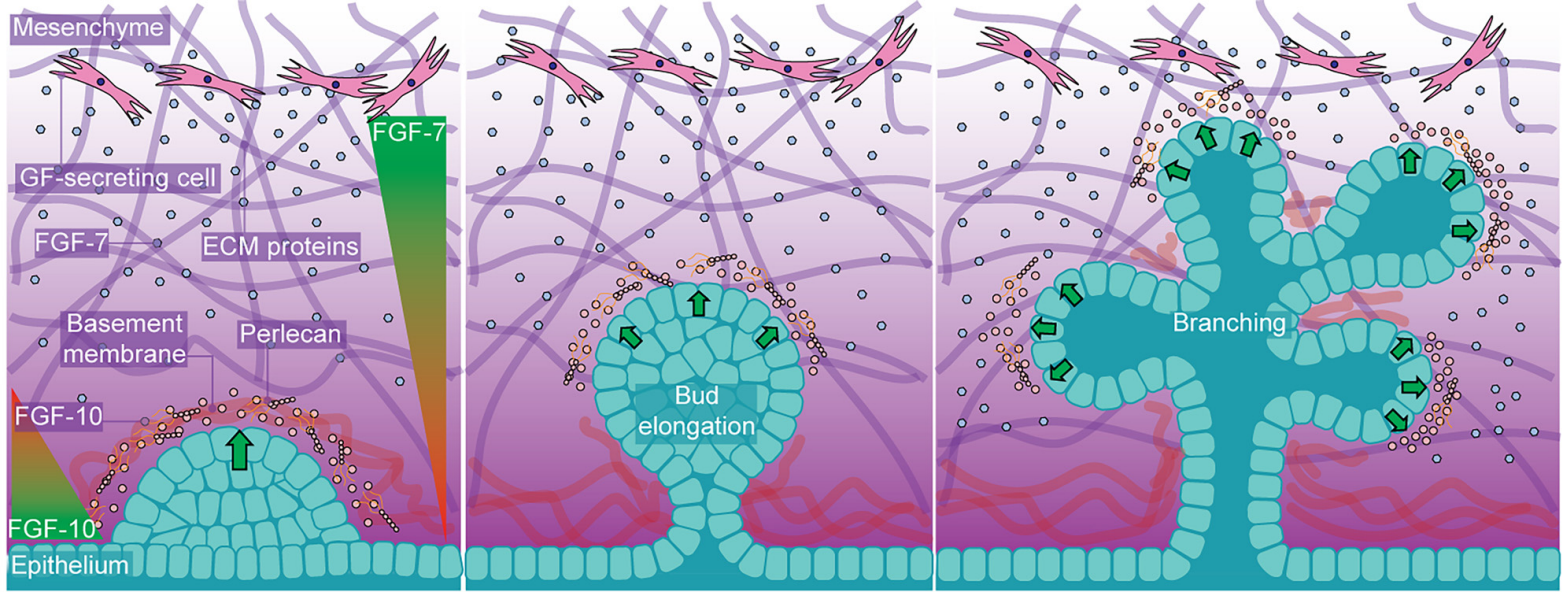
The role of HS in forming GF gradients in branching morphogenesis. HS plays an essential role in branching morphogenesis, forming tight gradients of FGF-10 that induce budding and bud elongation and diffuse gradients of FGF-7, which induce branching.

HS in the ECM acts as a GF reservoir, and numerous GFs are sequestered by perlecan in the pericellular matrix.^58^ When necessary, HS-bound GFs are released and transported to target cells, creating signaling cascades and stimulating the endogenous mechanisms of repair.^59–61^ Tissue and organ development are guided by the formation of GF diffusion gradients in the ECM,^62,63^ HSPGs play a central role in forming FGF gradients, which guide midbrain development (FGF-8).^64^ The affinity of interactions between different FGFs and HS also influences tissue development, particularly during branching morphogenesis (Fig. 3), and is an essential process during the development of the salivary and lacrimal glands. Low-affinity interactions with FGF-7 lead to diffuse gradients, which stimulate epithelial branching, whereas high-affinity interactions with FGF-10 create tight gradients that induce epithelial bud elongation.^65^ Mutations that perturb these interactions can alter diffusion gradients, highlighted by aberrant joint development in FGF-9 mutant mice.^66^ These studies highlight the essential role HS plays in forming morphogen gradients and how subtle variations in affinity toward signaling molecules can result in different biological outcomes.

### C. Release of HS-bound GFs

The release of HS-bound GFs occurs through changes in the local environment (e.g., temperature, acidity, and ionic strength) or enzymatic cleavage.^67^ Mechanical compression of tissues such as articular cartilage can deform the matrix, causing pericellular HSPG perlecan to present bound FGF-2 to cell surface FGFRs.^67^ This mechanism drives an immediate injury response and initiates the tissue repair process.^68^ A similar mechanism has been reported for TGF-β1.^22^ GFs are also released through enzymatic cleavage of HS chains by heparanase or release of cell surface HSPGs by Notum.^62^ Heparanase is a secreted HS-degrading endoglycosidase that is found in the ECM and at the cell surface.^69,70^ Increased levels of heparinase at wound sites facilitate the release of pro-regenerative GFs like FGF-2 and PDGF-BB, increasing bioavailability and diffusion and improving cell migration, proliferation, and neovascularisation.^71^ This mechanism has been further confirmed through exogenous application of a combination of heparanase, HS, and GFs to skin lesions in a mouse model, recapitulating the natural wound healing process.^72^ The release of FGF-10 from perlecan HS chains by exogenous recombinant heparanase enhances branching morphogenesis.^73^ Similarly, heparanase treatment influences BMP and Wnt signaling in hMSCs, enhancing osteogenic differentiation.^74,75^ HS can also negatively regulate GF activity by facilitating internalization and reducing its accessibility to the receptor, as reported for BMP-2 and FGF-2, highlighting the importance of HS in influencing the availability and bioactivity of HS-binding GFs within the ECM.^54,76^

### D. Leveraging HS interactions to improve GF delivery

Current exogenous GF delivery methods for tissue repair, such as direct injection into the target site, have limitations, including rapid diffusion, short-lived bioactivity, and a short half-life, necessitating a supraphysiological dose.^77^ Affinity toward HS allows for slower, controlled diffusion through tissues. Lactoferrin, which is similar in structure to transferrin, exhibits a 60% lower diffusion coefficient, owing to a cluster of basic amino acids at the N-terminus that enables interaction with HS.^78^ Engineering GFs to have increased binding affinity toward HS can enhance their activity by slowing diffusion and improving retention of GFs at the cell surface, leading to sustained signaling. Engineered variants of VEGF_165_ and PDGF-BB, containing high-affinity HS-binding peptide sequences derived from laminin subunit alpha-1, enhanced their binding to cell surface HS chains, in turn leading to sustained signaling through VEGFR2 and PDGFRβ, respectively.^79^ These studies highlight the importance of HS in modulating GF signaling gradients and sustaining GF signaling, properties that can be leveraged when developing regenerative therapies using HS-binding GFs.

Using HS–GF interactions as a strategy for GF delivery requires the immobilization of HS, or the highly sulfated analog heparin, onto a suitable carrier; otherwise, the HS–GF complex will rapidly diffuse. As previously discussed in Sec. II B, heparin is widely used as a substitute for HS in biomaterial applications due to greater availability and a similar structure, albeit with a much greater degree of sulfation. Multiple strategies, including both covalent and non-covalent approaches, exist, such as chemical or physical cross-linking, encapsulation, and ionic adsorption.

Modifying HS for covalent attachment can be performed using a variety of chemistries, owing to the abundance of reactive groups within the HS chain, including carboxylic acids, hydroxyls, amines, and aldehyde groups. Thiolation of heparin has been employed extensively for cross-linking into various materials, including hydrogel films.^80^ Thiolation and many other chemical cross-linking strategies rely on activating carboxylic acid groups using carbodiimide reagents, which allows for stable amide bond formation through reaction with amine-functionalized cross-linkers and other molecules.^81^ While straightforward and efficient, carbodiimide activation of HS and heparin is not site-specific due to the high abundance of carboxylic acid groups (one per disaccharide), which can result in steric hindrance and perturb function. HS and heparin may also be modified using reductive amination or oxime ligation to functionalize the reducing end aldehyde group, allowing for direct conjugation to a material in a natural orientation or introducing a single functional group, such as an azide, biotin, or cyclooctyne.^82,83^ A diverse range of materials, both synthetic and naturally derived, can be modified with HS and heparin, including collagen I, chitosan, polyethylene glycol, gelatin, and fibrin.^84–93^ These modified materials have been used for the sustained release of several GFs involved in tissue regeneration, including BMP-2,^59,94^ FGF-2,^95^ VEGF_165_,^95^ and IL-4.^57^

In addition to covalent attachment, the polyanionic nature of HS and heparin facilitates non-covalent immobilization via electrostatic interactions with cationic materials, including cationic peptides,^96^ positively charged collagen,^90^ or cationic polymers such as poly(allylamine).^97^

Together, these studies highlight the importance of HS as a natural storage molecule for GFs within the ECM. This property can be harnessed when developing novel materials with tunable GF release profiles, with the more widely available heparin serving as a substitute for HS in many recent materials. The behavior of HS and heparin-modified materials can be influenced by multiple factors, including temperature, pH, ionic strength, and degree of cross-linking.^95,98,99^ With the knowledge that these events are critical in development and wound healing, creating novel HS-functionalized materials designed to mimic an HS-rich ECM could improve GF delivery methods and enhance tissue regeneration.

## IV. THE ROLE OF HS IN GF:RECEPTOR INTERACTIONS AND SIGNALING CASCADES

### A. HS as a co-receptor

HSPGs influence GF availability to their cognate receptors by functioning as low-affinity co-receptors. HSPGs sequester and concentrate GFs at the cell surface and in the pericellular matrix, increasing the frequency of GF:receptor interactions. These interactions can confine GF diffusion within the HS-rich glycocalyx at the cell surface or function to translocate GFs through the ECM to nearby cells.^100^ HS may function as an essential part of an active signaling complex by binding receptor and ligand (such as FGF–FGFR)^101,102^ or serve to recruit receptor and ligand to an active signaling complex (such as BMP-2–BMPR2).^103^ Upon binding, GF–receptor complexes form and signaling pathways are activated, which tightly regulate gene expression and key biological functions, including proliferation, migration, and differentiation. Importantly, HS can function as a co-receptor at the surface of an individual cell, across adjacent cells, or when part of a matrix HSPG. Cell surface HS chains contribute to active signaling complexes on neighboring cells, highlighted in a study by Jakobsson *et al.* using chimeric embryoid bodies formed of *Vegfr-2^−/−^* and *Ndst1/2^−/−^* murine embryonic stem cells.^104^ Here, VEGF signaling of endothelial cells defective in HS production can be fully supported by HS from adjacent perivascular smooth muscle cells, defective in VEGFR2. ECM-associated HS has also been shown to participate in active signaling complexes; perlecan-bound HS can interact with FGF-18 and FGFR3 to form ternary complexes during cartilage development.^105^

### B. FGF signaling

The role of HS in the FGF signaling pathway is perhaps the quintessential example of the role of HSPGs as co-receptors, as HS forms an integral part of multiple FGF:FGFR signaling complexes.^102,106^ The FGFs are a family of 23 heparin-binding growth factors with tertiary structures of 12 antiparallel β-strands arranged in a threefold internal symmetry. Dependent upon their interactions with HS, FGFs can be categorized into the major paracrine subfamilies (such as FGF-1 and FGF-2) and the minor endocrine subfamilies (e.g., FGF-19, FGF-21, and FGF-23); the activity of paracrine FGFs is mainly dependent on HS binding.^107^ Endocrine FGFs have substantially weakened HS-binding affinities, a property that facilitates diffusion into the bloodstream to reach distant targets.^108^ The molecular basis of the interactions indicates a degree of selectivity in FGF–heparin/HS interactions and FGF–FGFR interactions with heparin/HS as a co-receptor.^25,109^ Biochemical studies show HS acts as a ‘molecular glue’ connecting two paracrine 1:1 FGF–FGFR complexes to construct a 2:2:2 FGF–FGFR–HS dimeric signal transduction unit on the cell surface.^110^ Other FGF–FGFR–HS ternary complex binding modes have been proposed, including a 2:2:1 ratio.^111–113^ Once tyrosine kinase receptors (FGFRs 1–4) are activated, the transphosphorylation of intracellular kinase domains occurs, and kinase activity is upregulated, resulting in the phosphorylation of downstream signaling molecules.^107^ Through x-ray diffraction analysis of crystal complex structures, three peptide loops in HS-binding sites have been identified, which are responsible for reinforcing FGF–FGFR proximity. These loop regions exhibit similar topologies but minor sequence differences, which may explain the preferences for different sulfation patterns in HS.^16^ FGF-1 and FGF-2 display different preferences for sulfation patterns when binding HS; however, FGF–FGFR–HS complex formation is enhanced by increasing sulfation, regardless of the specific location of the sulfate groups.^114,115^ More recent studies have suggested the FGF-1–FGFR1c complex requires longer sulfated domains than FGF-2–FGFR1c complexes.^116^ Specific sulfate groups have also been shown to enhance activity of smaller HS and heparin oligosaccharides, with 2-*O*-sulfation at the non-reducing end of short oligosaccharides (dp4–dp6) enhancing the mitogenic activity of FGF-2.^117^ Even disaccharides have been shown to induce transient FGF signaling, though longer oligosaccharides (dp4 and above) are required to sustain signaling.^118^

### C. VEGF signaling

HS plays a key role in angiogenesis through interactions with the VEGF signaling pathway. In adults, the vasculature is relatively quiescent. VEGF stimulates endothelial cells during wound healing and tissue repair, promoting angiogenesis by enhancing cell migration and proliferation. VEGF_165_, one of the most common VEGF isoforms, binds HS co-receptors to potentiate angiogenic signals through receptor tyrosine kinases VEGFR1/2, leading to capillary development.^119^ *In vitro* studies show endothelial cells depleted of HS show poor VEGF_165_ activity, which can be partially restored by adding exogenous heparin or HS, highlighting the importance of HS for VEGF_165_ binding and signaling.^40,120^ HS:GF interactions are dynamic; HS-binding proteins with higher affinity can displace those with lower affinity from HS chains, affecting cell signaling. CXCL9, a potent GAG binder, could displace VEGF_165_ and FGF-2, resulting in significantly reduced signaling and angiogenesis.^121^

### D. BMP signaling

BMPs are members of the TGF-β superfamily and play a crucial role in bone development and various other developmental processes. Some BMP family members possess a high-affinity HS-binding domain, endowing HS with regulatory roles in BMP and BMPR signaling.^122,123^ In BMP-2-responsive C2C12 cells, BMP-2-induced ALP activity can be enhanced approximately threefold by HS.^54,55^ However, the relationships between BMP signaling and HS remain unclear, sometimes producing contradictory results. Kuo *et al.*^103^ reported that heparitinase treatment diminished exogenous BMP signaling, proposing that HS potentiates BMP activity by recruiting BMP type II receptor subunits to type I receptor complexes, acting as a catalyst rather than a co-receptor. In contrast, others have reported heparitinase treatment increases BMP availability and enhances BMP signaling by removing cell surface HS. This suggests HS may compete with BMP receptors for interaction with BMP ligands, limiting BMP-2:BMPR interactions and consequently reducing BMP-2-mediated mineralization.^75,124^ Inhibition of cell surface HS using Surfen has also been shown to increase BMP-2 signaling, suggesting the bone nodule growth observed in hereditary multiple exostoses may be a result of the loss of HS, resulting in overactive BMP-2 signaling.^125^ Beyond binding BMPs, HS can prevent the inhibition of BMP-2 activity by BMP antagonist noggin.^55^ HS may also facilitate the internalization and degradation of BMPs.^103,124,126^ These contrasting observations suggest that HS plays a multifaceted role in BMP signaling, which the composition, concentration, and location of HS may influence.

## V. HS AS A REGULATOR OF CELL AND TISSUE FUNCTION

Cell surface or matrix-bound HS is essential for maintaining tissue integrity and homeostasis. At the cellular level, HS serves as a key regulator of cell signaling,^127,128^ adhesion,^129^ morphogenesis,^130^ migration,^61^ proliferation,^131^ and fate decisions.^132^ HS regulates these complex cellular responses via interactions with a myriad of ligands, including GFs, cytokines, and chemokines, while also participating in cell–cell and cell–matrix interactions that influence tissue architecture and organization.^133,134^

### A. Inflammation

Beyond the localization on cell surfaces and within the ECM, HSPGs can also be found distributed throughout the blood vessel wall as part of the endothelial glycocalyx, where they exert the spatiotemporal regulation of cellular responses to injury and inflammation.^133,135^ The inflammatory response involves the swift recruitment of leukocytes, such as neutrophils and monocytes, from the bloodstream to crawl along the endothelium toward the site of inflammation by an immobilized gradient of chemokines, a process called haptotaxis or chemotaxis.^136^ More than 50 chemokines have been identified, and all are known or predicted to bind to HS.^137,138^ HSPGs are responsible for transporting these chemokines, such as IL-8, from perivascular cells to the luminal surface of the endothelium via transcytosis.^139,140^ This creates a concentration gradient that guides leukocyte migration by anchoring chemokines, preventing them from being carried away by the blood flow. HS also plays an important role in leukocyte rolling and movement arrest. Leukocyte L-selectin binds HS in the endothelial glycocalyx, slowing leukocyte rolling velocity, eventually resulting in arrest, firm attachment, and migration.^139^ Cytokine-activated leukocytes and endothelial cells produce heparanase, which degrades the cell surface HS, facilitating the transmigration of leukocytes through the endothelial lumen into surrounding tissues.^141^ Soluble HS fragments released during inflammation are recognized by Toll-like receptor 4 (TLR4),^142^ a key activator of the innate immune response, which results in the maturation of antigen-presenting cells such as dendritic cells^143^ and macrophages.^144^ It is becoming increasingly evident that HS plays multiple roles in immune regulation and signaling during tissue injury and inflammation, both in the microenvironment and on the cell surface of leukocytes. This makes HS an attractive candidate for use in immunomodulatory and anti-inflammatory applications.

### B. Vasculature

HS is expressed abundantly in both developing and mature vasculature. The balance between pro- and anti-angiogenic factors intricately regulates vascular formation.^145^ Disruptions in angiogenesis and the vasculature are implicated in various conditions, including malignancies, inflammatory diseases, ischemic events, infections, and immune-related disorders. HS has been shown to bind and potentiate both pro- and anti-angiogenic factors.^146^ Genetic knockouts of genes encoding HS biosynthetic enzymes [including 6-*O*-sulfotransferase 1 (*Hs6st-1*), 3-*O*-sulfotransferase 1 (*Hs3st-1*), *N*-deacetylase/*N*-sulfotransferase 1 (*Ndst-1*), C5-epimerase (*HSepi*), and exostosin 1 and 2 (*Ext-1 and Ext-2*)] in animal models have demonstrated both the broad functions and the intricate structural roles of HS in regulating vascular development under conditions that closely mimic those in humans.^146,147^ Genetic manipulation of *Ndst* expression enables the study of the role of HS sulfation in regulating the function of vascular endothelial cells. Loss of *Ndst-1* in mice limits PDGF-BB binding, which is critical for pericyte migration and attachment during vascular development. This impairment leads to inadequate spreading and tight attachment of vascular smooth muscle cells (VSMCs) during vascular maturation.^148^ This was also confirmed in a mouse model lacking smooth muscle *Ndst1*, where a significant decrease in proliferating VSMCs, reduced vessel size, and a substantial reduction in lesion formation were observed in response to vascular injury,^149^ suggesting that alterations in HS structure can affect vascular structure and remodeling. Total loss of HS by knockout of *Ext-1* also results in defects in pericyte attachment, aberrant angiogenesis, PDGFB signaling, and TGF-β signaling.^150^ Reduced endothelial cell expression of HS6ST-1 or HS6ST-2 has been shown to diminish FGF-2- and VEGF_165_-mediated angiogenesis, thereby adversely affecting endothelial cell function and phenotype.^151^ Interestingly, silencing of endothelial *Ext-1* and *Ext-2* with small interfering RNA significantly improves nitric oxide synthesis and maximizes arteriolar dilation during reperfusion.^147^ These studies have demonstrated that HS is critical in modulating angiogenesis and maintaining endothelial function and hemostasis. This regulatory role depends significantly on the fine structure of HS, which facilitates the binding of various GFs to the cell surface.

### C. Angiogenesis

**FIG. 4.**
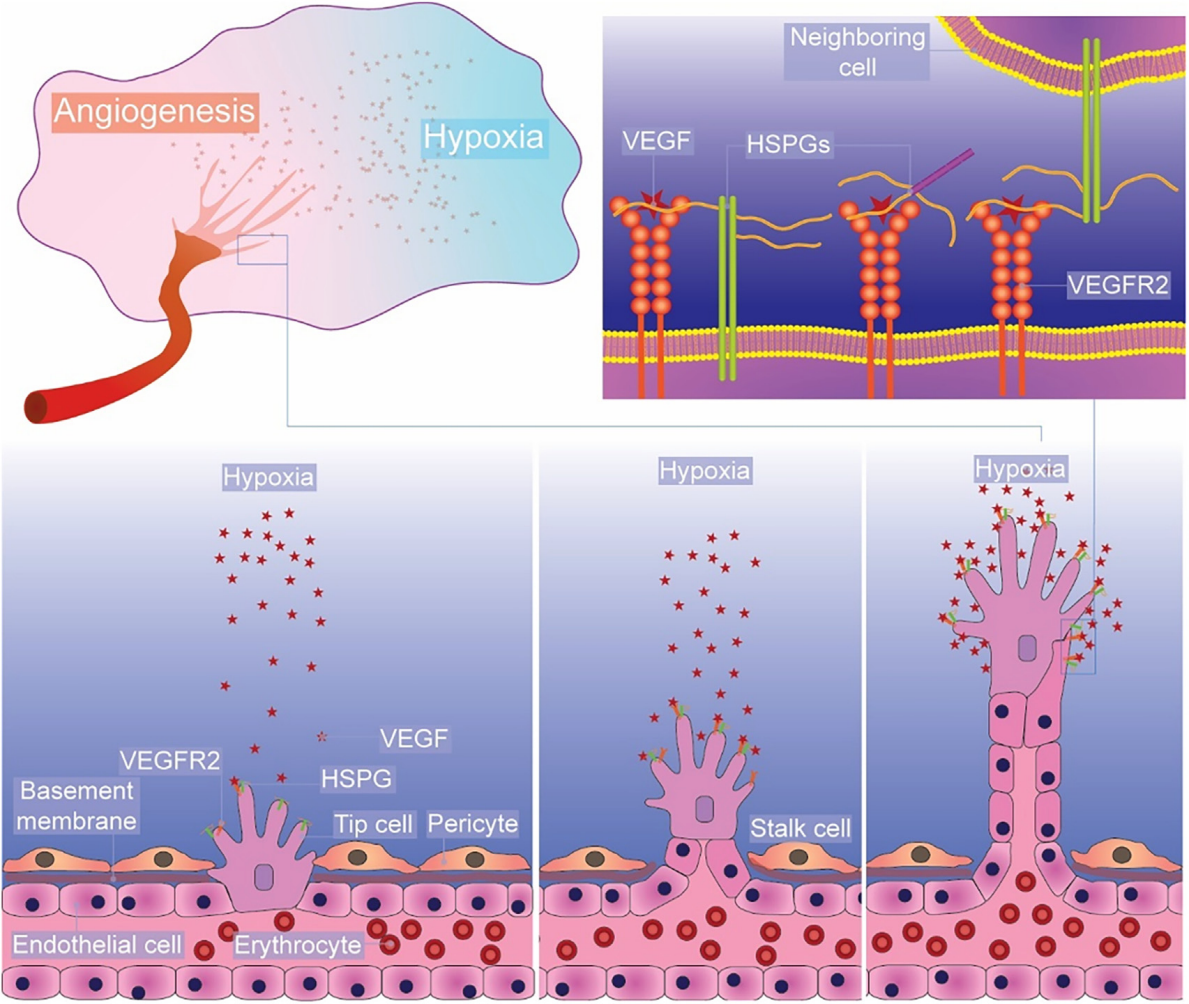
Cell surface HSPGs regulate angiogenesis. Hypoxia-induced expression of VEGF_165_ leads to angiogenic sprouting and the growth of new vasculature. HSPGs act as key co-receptors in VEGF_165_ signaling, sequestering VEGF at the cell surface and mediating VEGF–VEGFR2 signaling complexes. HSPGs at the cell surface, in the pericellular matrix, and on neighboring cells can all contribute to VEGF–VEGFR2 complex formation.

Angiogenesis is vital for tissue repair and regeneration. Chiodelli *et al.*^152^ summarized the interactions between angiogenic modulators (including angiogenic GFs, pro-angiogenic receptors, and angiogenic inhibitors) and HS/heparin. VEGF_165_ is one of the most potent pro-angiogenic GFs, serving as a key player in promoting this process during wound healing.^153^ As previously mentioned in Sec. IV C, HS binds VEGF_165_, increasing its bioavailability and enhancing VEGFR2 signaling (Fig. 4). HS can also restore binding between oxidized VEGF_165_ and VEGFR2.^154^ This function is essential in hypoxic environments, such as wounds, where new vessel formation is a prerequisite for tissue repair and regeneration. A murine model of HS-deficient perlecan (*Hspg2*^Δ3/Δ3^) exhibited impaired FGF-2-mediated corneal angiogenesis and delayed wound healing, highlighting the indispensable role of HS in sustaining GF signaling necessary for wound repair.^155^

### D. Muscle regeneration

HS also plays an essential role in skeletal muscle regeneration. *In vivo* studies have demonstrated that HS significantly impacts muscle fiber thickness, muscle strength, and regeneration by promoting myogenic differentiation, all of which contribute to the maintenance of motor activity.^156,157^ Removal of HS via CRISPR–Cas9-mediated knockout of *Ext-1* or heparitinase treatment dramatically impaired myoblast differentiation of murine C2C12 cells and attenuated post-injury muscle regeneration in skeletal muscle-specific *Ext-1* knockout mice.^156^ Genetic ablation of 6-*O*-endosulfatases (*Sulf-1* and *Sulf-2*) in mice also impairs the function of muscle satellite cells.^157^ Double *Sulf-1/2* knockout mice display delayed myogenic differentiation after injury, yet single *Sulf-1* or *Sulf-2* knockout mice regenerate normally. This suggests that dynamic compositional changes in HS are crucial for tissue repair.^157^ *Sulf-1* and *Sulf-2* have also been identified as repressors of non-canonical Wnt signaling to promote myoblast fusion during skeletal muscle regeneration, possibly through the promotion of canonical Wnt signaling, which antagonizes non-canonical pathways.^158^ Changes in HS composition have also been associated with muscle aging. In a murine model, Ghadiali *et al.* observed an increase in HS 6-*O*-sulfation in aged muscle, promoting FGF-2 signaling and myoblast proliferation, which eventually led to satellite cell hyperactivation and consequent exhaustion.^159^

### E. Bone

HS is also known to modulate numerous pathways associated with bone formation and repair. Overexpression of syndecan-2 in a transgenic mouse model increased bone mass and GAG content at the bone surface. This study suggested osteoblastic HS controls bone remodeling by modulating Wnt signaling and regulating the production of Wnt effectors, which correlates with our previous findings that heparin can enhance osteogenesis by modulating Wnt3a signaling.^160,161^ Osteoblast HSPGs create a supportive microenvironment in the bone marrow, influencing other cell types such as MSCs. MSCs play a vital role in bone regeneration by differentiating into osteoblasts, the cells responsible for new bone formation. HS is also involved in BMP-mediated differentiation of MSCs into osteoblasts and bone matrix formation, contributing to the formation of mineralized bone tissue, structural stability, and tissue function.^75^ The knockout of perlecan in mice reduced the pericellular area and integrity of the osteocyte lacunocanalicular system, which allows cell-to-cell communication and fluid exchange throughout the calcified bone matrix.^162^

## VI. APPLICATIONS OF HS IN REGENERATIVE MEDICINE

**FIG. 5.**
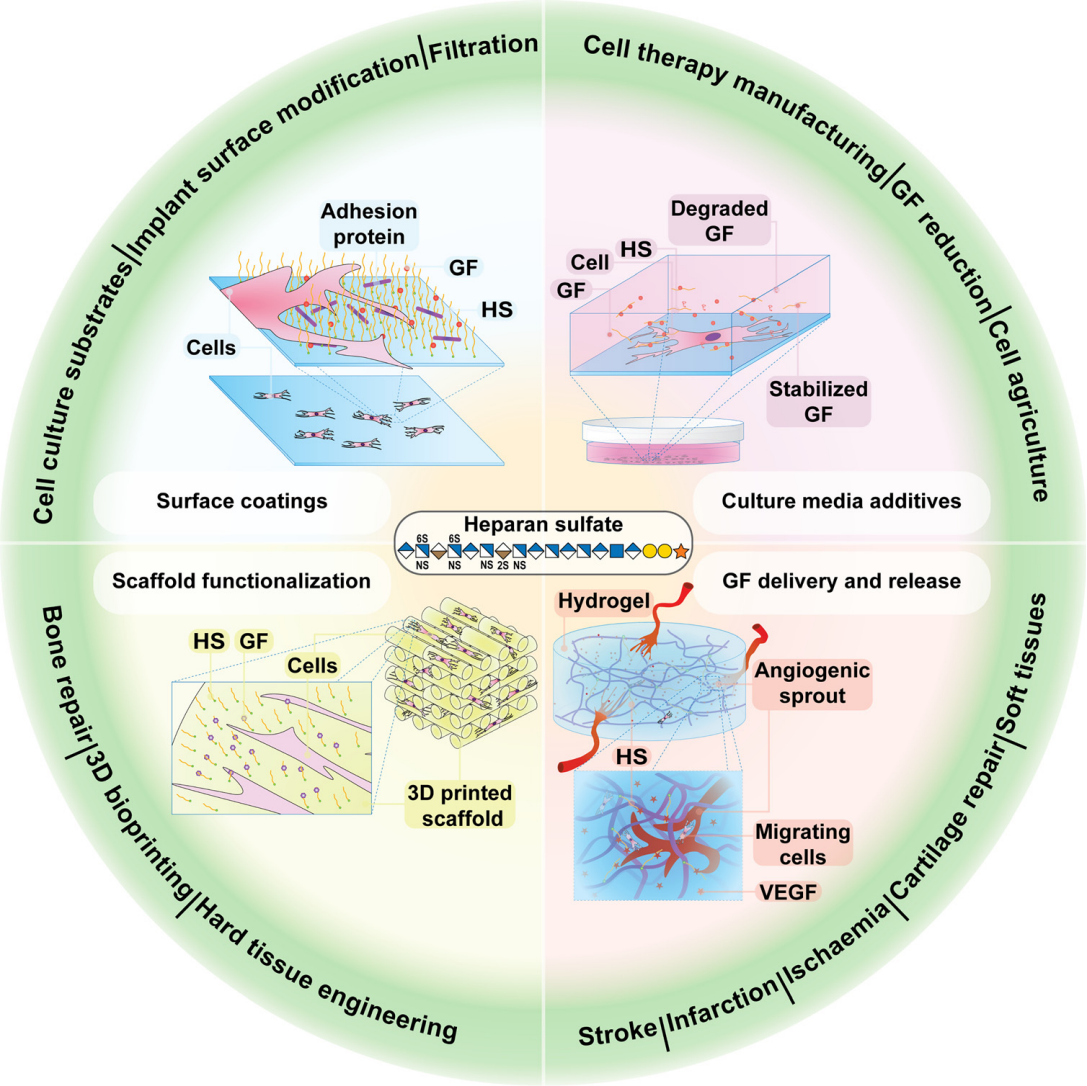
Applications of HS in tissue engineering and regenerative medicine. The multifaceted role of HS in biological processes has enabled its use in multiple therapeutic applications, including as a surface coating, cell culture media additive, component of hydrogels, and part of 3D scaffolds. The monosaccharides represented in this figure are derived from the symbol nomenclature for glycans (SNFG).^11^

Heparin has long been one of the most widely used GAGs for functionalizing biomaterials due to its availability, well-established safety profile, and regulatory approval. In the past decade, more studies have begun using HS in similar biomaterial applications, given HS, not heparin, is a key component of the ECM. This property makes HS-based biomaterials an ideal choice for various tissue engineering applications (Fig. 5), providing the optimal means to modulate GF activity at physiological concentrations and influence cell behavior in different tissues. This represents a conceptual shift in GF use in tissue engineering, moving away from the current approach of using supraphysiological GF concentrations to one that minimizes or removes exogenous GFs, resulting in improved and more successful therapeutic outcomes.^163^

### A. Neural tissues

Several studies have described the application of HS-functionalized biomaterials in neural tissues. For example, the functionalization of a 3D hydrogel with synthetic HS tetrasaccharides can transiently stabilize the FGF-2–FGFR1 complex, modulating the proliferation and neuronal differentiation of human neural stem cells (NSCs).^164^ NSC proliferation was similarly supported by collagen/HS porous scaffolds, which enhanced regeneration and restored neurological function when implanted into a rat model of traumatic brain injury.^165^ These studies highlight the potential of HS-functionalized biomaterials for providing a supportive microenvironment for NSCs, which leads to improved tissue repair. We have demonstrated that direct intracerebroventricular injection of a VEGF-binding HS variant, termed HS7, can enhance vasculogenesis and neurogenesis in a rat model of cerebral ischemia, resulting in improved post-ischemic recovery. This suggests that HS can enhance tissue regeneration without the need for a carrier. Direct intramuscular injection of HS7 into rodent models of hindlimb ischemia resulted in functional improvements in angiogenesis, blood flow, and hindlimb function, further highlighting the potential of HS to enhance the activity of endogenous HS-binding GFs.^4,166^

### B. Bone repair

HS has also been used extensively in orthopedic applications to enhance bone regeneration. We have previously developed an affinity-isolated BMP-2-binding HS variant, termed HS3, and employed it in several animal models of bone repair using various biomaterials. Our initial study on HS3 resulted in significant structural and functional improvements in a critical-sized rabbit ulnar defect.^55^ We have also successfully combined HS3 with an FDA-approved β-tricalcium phosphate bone graft substitute (JAX™) in the same animal model, with the addition of HS3 significantly improving bone formation.^167^ HS3 is also capable of enhancing bone regeneration in periodontal defects. When passively absorbed into a cross-linked collagen I scaffold, HS3 was able to improve periodontal bone regeneration, and when combined with BMP-2, resulted in a fourfold increase in new alveolar bone formation and a ∼1.5-fold improvement in functional ligament restoration.^168^ Polycaprolactone–hydroxyapatite (PCL–HA) porous 3D scaffolds loaded with HS have also been shown to promote osteoblast maturation and accelerate bone defect repair in both *in vitro* and *in vivo* studies.^46,47,55,167^ When incorporating HS into medical-grade implants and materials, it is essential to understand if the function of HS can be preserved after medical-grade sterilization, the gold standard of which is a 25 kGy dose of gamma irradiation. We reported that the structure and composition of HS3 remained unchanged when subjected to a 25 kGy dose of gamma irradiation, thereby advocating for the feasibility of incorporating HS into medical-grade implants.^56^ More recently, Duan *et al.* developed a 3D-printed composite PCL/gelatin/HS scaffold with a vascular-like hierarchical structure loaded with a PDGF-BB–LG4 fusion protein. When implanted into a critical-sized calvaria defect, the scaffold enhanced bone regeneration through improved osteogenesis and angiogenesis, offering significant improvements over PCL/gelatin scaffolds alone.^169^ This study again highlights the potential of HS-based scaffolds for enhancing GF delivery and tissue regeneration.

### C. Enhancing cell growth and proliferation

We have previously developed an affinity-isolation platform using peptides analogous to the heparin-binding domains of several GFs. This has allowed us to fractionate novel HS variants with improved affinity for specific GFs, such as HS8, an HS variant with high affinity toward FGF-2.^48^ We have demonstrated that HS8 can enhance the stability of FGF-2, prolonging FRS2α and ERK1/2 phosphorylation and accelerating the expansion of MSCs without compromising their potency. HS8-expanded MSCs improved the healing of osteochondral defects using multiple animal models.^45^ More recently, highly sulfated recombinant HS combined with collagen I has been shown to reduce the need for exogenous GFs while effectively supporting hMSC growth and enhancing anti-inflammatory potency in both planar- and microcarrier-based cultures, even with minimal GF supplementation.^170^

### D. Using HS mimetics as regenerative therapeutics

HS analogues that closely mimic the structure or charge density of native HS are tailored to promote repair and regeneration in various tissue lesions. These targeted HS mimetics minimize the adverse side effects typically associated with the nonspecific binding interactions of HS with clotting factors and other biochemicals present in the tissue microenvironment.^171^ Sheng *et al.*^172^ developed a new class of HS mimetics with defined sulfation motifs and the ability to inhibit pro-inflammatory chemokines with similar efficiency to natural heparin but did not interact with key factors in the coagulation cascade. Administration of HS mimetic OTR4120 significantly reduced diabetic ulcer healing time and improved healing quality by increasing angiogenesis and reducing inflammation and collagen type III synthesis, which is often associated with excessive scarring during tissue remodeling.^173,174^ Local injection of a family of HS mimetics called RGTAs^®^ (ReGeneraTing Agents)^175^ showed accelerated postinjury skeletal muscle regeneration by stimulating both myogenesis and angiogenesis.^176,177^ More recently, HS mimetics have also been considered promising agents for bone and periodontal regeneration.^178,179^ These mimetics predominantly function by augmenting endogenous BMP activity, promoting osteogenesis and bone regeneration.^180–182^

Designing HS mimetics with specific sulfation patterns to precisely mimic natural HS interactions with various GFs could enhance their efficacy and minimize off-target effects.^182^ A deeper understanding of the molecular mechanisms by which HS interacts with proteins to promote tissue regeneration will guide the creation of defined, targeted HS variants. This knowledge can also inform the synthesis of HS mimetics of defined structure, offering a cost-effective alternative to natural sources for targeting critical pathways in tissue regeneration.

## VII. CHALLENGES AND PERSPECTIVES

GF therapies are an attractive strategy for enhancing tissue repair. However, due to the drawbacks mentioned in this perspective, there is a strong need to develop novel materials for more efficacious GF delivery. Over the last 30 years, the essential roles of HS in modulating GF activity have been elucidated. This includes enhancing GF stability, attenuating degradation, modulating extracellular diffusion and internalization, enhancing tonic cellular signaling, and directing cell behavior, ultimately promoting cell proliferation, differentiation, and tissue development. Until recently, HS-based biomaterials had yet to undergo clinical assessment,^183^ largely due to the complex and heterogeneous structure of HS and the many biological functions it fulfills. Several challenges remain to be addressed moving forward.

GFs exhibit differential affinities to HS, necessitating precise regulation of HS concentration to balance signaling pathways for positive responses. Mundy *et al.*^125^ suggested that HS deficiency may potentially reduce FGF-dependent anti-chondrogenic signaling and reciprocally enhance BMP-dependent pro-chondrogenic activity. When exogenous HS chains are present in high abundance, they outcompete receptors and cell surface HS for binding, leading to suppressed proliferation and migration.^60^ These studies highlight the need to optimize the appropriate HS concentration to effectively leverage GF activity and maximize performance, promoting interactions with receptors and resulting in target gene expression, which produces the desired reparative effect.

The vast number of potential sequences within different HS chains diversifies their biological functions. HS with different structural characteristics has been found to exert variable effects on the bioactivity of VEGF_165_. Administration of high molecular weight heparin promotes VEGF_165_-mediated angiogenesis by elongating microvessel sprouts, while low molecular weight heparin significantly inhibits VEGF_165_ binding to its receptors and shortens microvessel sprouts.^152^ HS/heparin is also capable of inhibiting FGF-2 signaling. Exogenous heparin and some heparin octasaccharides with distinctive structures may compete with endogenous HS proteoglycan and impede the formation of the FGF-2–FGFR1–HS ternary complex.^184^ These studies highlight the complex and varied role HS plays in regulating GF signaling and the continuing need to study the structure–function relationships between HS and HS-binding proteins.

One challenge in translating HS-based therapeutics into the clinic is preparing pure, well-characterized HS materials at a sufficient scale. HS is the most heterogeneous GAG, with a diverse tissue- and cell-specific composition, molecular weight, and other structural characteristics, making it a challenging molecule to characterize fully.^185^ There is a strong need to produce highly purified and structurally defined HS variants, which will also help to elucidate HS:GF structure:function relationships by serving as well-defined standards of known structure and composition.^186^

Tissue repair requires simultaneous or sequential delivery of multiple GFs over time. For example, angiogenesis requires VEGF, FGF, and angiopoietin-2 (Ang-2) to form new immature vessels and angiopoietin-1 and PDGF-BB to stabilize these newly formed blood vessels.^187^ Co-delivery of FGF-2, TGF-β1, and adipose-derived MSCs significantly improved the healing of critical-sized femoral defects in a mouse model.^188^ It is essential to comprehensively decode interactions between HS and GFs in a co-delivery system to ensure effective improvement of tissue regeneration. We foresee the development of tailor-made HS-based therapeutics with distinct GF-binding profiles and release kinetics, which can potentiate appropriate cellular responses to induce tissue regeneration facilitated by the emergence of innovative techniques in the future.

## VIII. CONCLUDING REMARKS

Throughout this perspective, we have discussed the roles that HS plays in orchestrating tissue repair through its interactions with numerous GFs, signaling molecules, and receptors. These properties can be exploited to develop novel solutions for regenerative medicine, including materials for GF delivery, stabilization, and release. Developing HS variants of defined structure and composition through metabolic engineering, chemoenzymatic synthesis, or chemical synthesis offers a promising pathway toward recapitulating the biological functions of HS in a controlled manner. These novel variants could be used as GF stabilizers to reduce the costs of cell culture media, bioactive epitopes in biomimetic materials for the controlled release of GFs for tissue repair, or as coatings on medical devices to improve biocompatibility and cell attachment. HS displays great promise as a modulator of GF dynamics in numerous tissue regeneration applications, highlighting its potential as a bioactive component in novel biomaterials for clinical translation.

## Data Availability

Data sharing is not applicable to this article as no new data were created or analyzed in this study.
